# RNA Silencing Is Resistant to Low-Temperature in Grapevine

**DOI:** 10.1371/journal.pone.0082652

**Published:** 2013-12-20

**Authors:** Marjorie Romon, Isabelle Soustre-Gacougnolle, Carine Schmitt, Mireille Perrin, Yannick Burdloff, Elodie Chevalier, Jérome Mutterer, Christophe Himber, Jérôme Zervudacki, Thomas Montavon, Aude Zimmermann, Taline Elmayan, Hervé Vaucheret, Patrice Dunoyer, Jean E. Masson

**Affiliations:** 1 UMR1131 Santé de la Vigne et Qualité du Vin, INRA-Université de Strasbourg, Colmar, France; 2 Laboratoire Vigne, Biotechnologies et Environnement, Université de Haute Alsace, Colmar, France; 3 Institut de Biologie Moléculaire des Plantes, UPR2357, CNRS, Strasbourg, France; 4 Institut Jean-Pierre Bourgin, UMR1318, INRA-AgroParisTech, Versailles, France; CNRS UMR7622 & University Paris 6 Pierre-et-Marie-Curie, France

## Abstract

RNA silencing is a natural defence mechanism against viruses in plants, and transgenes expressing viral RNA-derived sequences were previously shown to confer silencing-based enhanced resistance against the cognate virus in several species. However, RNA silencing was shown to dysfunction at low temperatures in several species, questioning the relevance of this strategy in perennial plants such as grapevines, which are often exposed to low temperatures during the winter season. Here, we show that inverted-repeat (IR) constructs trigger a highly efficient silencing reaction in all somatic tissues in grapevines. Similarly to other plant species, IR-derived siRNAs trigger production of secondary transitive siRNAs. However, and in sharp contrast to other species tested to date where RNA silencing is hindered at low temperature, this process remained active in grapevine cultivated at 4°C. Consistently, siRNA levels remained steady in grapevines cultivated between 26°C and 4°C, whereas they are severely decreased in *Arabidopsis* grown at 15°C and almost undetectable at 4°C. Altogether, these results demonstrate that RNA silencing operates in grapevine in a conserved manner but is resistant to far lower temperatures than ever described in other species.

## Introduction

Grapevine is cultivated worldwide on 8 M ha. Almost all acreage consists in vinifera scions, which give rise to qualitative grapes for food or wines, grafted on rootstocks. These rootstocks are complex hybrids between wild *Vitis* species and *Vitis vinifera*, which were mostly selected for resistance to phylloxera, but also adaptive criteria to specific soil composition. For example, the champagne is produced from bunches of *V. vinifera* cv. Pinot Noir, Pinot Meunier and Chardonnay, which are highly heterozygous accessions vegetatively propagated since centuries [1], [2]. These scions are grafted on the few rootstocks adapted to the characteristic limestone soils of the Champagne areas, the 41B and SO4, mainly. Vineyards and wild species, in their natural habitat, are hosting a wide collection of different viruses, among which the *Grapevine fanleaf virus* (GFLV) and *Grapevine leafroll-associated virus* (GLRaV) series are the most damaging [3]. Therefore, grape breeders thought that combining highly valuable rootstocks with virus-resistance would help improving grapevine culture. However, the quest for natural resistance to these viruses has failed until now.

RNA silencing is a natural defence mechanism against invading viruses in plants, and entails the production, from double-stranded (ds)RNA precursors, of virus-derived small interfering RNAs (siRNAs) by DICER-LIKE enzymes. Upon their incorporation into specific Argonaute (AGO)-containing effector complexes, these siRNAs guide post-transcriptional gene silencing (PTGS) of the viral genomes through RNA cleavage [4]. In plants, RNA silencing can be experimentally induced by transgene loci expressing inverted-repeat constructs (IR-PTGS) [5], or by highly transcribed sense transgenes (S-PTGS) [6], [7]. In this latter case, the dsRNA precursors are produced through the action of the endogenous RNA-DEPENDENT RNA POLYMERASE6 (RDR6), which converts single-stranded RNA into dsRNA. Importantly, if the transgene that undergoes PTGS carries viral sequences, the plant becomes resistant to infection by the homologous virus because both transgene mRNA and viral RNA are targeted by the transgene-derived siRNA. This strategy initially coined pathogen-derived resistance, was shown to confer resistance in a large number of species [8], [9]. With respect to grape, generating silencing-based virus-resistant transgenic rootstock seems a particularly adapted strategy. Indeed, given that RNA silencing in plants functions in a non-cell autonomous manner and has the potential to propagate from rootstocks to scions [10], [11], this approach would preserve the genetic complexity of the scion material which gives rise to bunches while conferring them enhanced resistance.

However, analyses of the effect of temperature changes previously revealed that RNA silencing is inhibited at low temperatures in Arabidopsis, *N. Benthamiana*, potato, *N. Tabacum*, soybean and petunia [12]–[15], questioning the relevance of the pathogen-derived resistance strategy in perennial plants such as grapevines, which are often exposed to low temperatures during the winter season. Therefore, as a first step towards implementing a strategy to obtain virus-resistant transgenic grapevines, we addressed the efficiency of IR-PTGS against a transgene in grapevines exposed to low temperatures. To do so, we developed an efficient transformation procedure on the sequenced PN40024 accession, which derived from a near homozygous inbreeded Pinot Noir line obtained by successive selfings [16]. By generating several stable transgenic Vitis lines, expressing GFP either alone or in combination with an IR construct, we show that IR-PTGS in grapevine, like previously observed in other plant species, triggers a very efficient silencing of the targeted mRNA in most somatic tissues, but is impaired in meristematic cells. IR-derived dsRNA in grapevine is processed into 21 nt- and 24 nt-long siRNAs, which trigger the degradation of the targeted GFP mRNAs and the subsequent production of 21 nt-long secondary siRNAs, most likely through the action of RDR6. Interestingly, and in sharp contrast with all plant species tested so far where RNA silencing is strongly impaired when temperature is lower than 15°C [12], the efficiency of IR-PTGS and the production of secondary siRNAs remain steady in grapevine cultivated at temperatures as low as 4°C, whereas they are severely decreased in *Arabidopsis* plants grown at 15°C and almost undetectable at 4°C. Altogether, these results demonstrate the efficiency of IR-PTGS in grapevine and show that, in this species, RNA silencing is resistant to far lower temperatures than ever described.

## Materials and Methods

### Plant Material, Initiation of Embryogenic Callus and Plant Regeneration

Pinot-derived line 40024 plants are growing in the field, in our repository. Collection of immature inflorescences and all steps from embryogenic callus (EC) induction to plant regeneration were conducted following described protocols [17], [18]. After 3 weeks on B medium, however, EC from the line 40024 were cultured on modified WPM (BAP 0.25 mg/l and 2.4D 1 mg/l and activated charcoal 3 g/l) for one month, and then transferred onto modified MPM1 (BAP 0.05 mg/l, 2.4 D 0.3 mg/l) for two 3-weeks periods. MPM01 medium [17] was used for EC long term maintenance. Control and transgenic grape plantlets were grown in vitro on WPM [26] at 26°C±0.5°C, 80%RH (Relative humidity), 16 h light at 2500 lux (Osram, Biolux) and 8 h dark at 25°C±0.5°C. Arabidopsis seeds (Col0) and GFFG/GFP line [19] were germinated on modified MS medium [20] in the same growth conditions than grapevine until cotyledon-first leaf developmental stage and then, transferred to the different temperatures regimes with the grape plantlets.

### Agrobacterium-mediated Transformation

Friable EC were sub-cultured at three-week intervals on MPM01 medium. Transformation with *Agrobacterium* strain C58pMP90 was performed two days after EC transfer onto fresh medium. *Agrobacterium* used harboured either the pBIN19-GFP (which confers kanamycin resistance) or the pFGC5941-GFFG (which confers BASTA resistance) binary vectors, and were previously described (24).The Agrobacterium was maintained on YEB (Yeast extract broth) solid medium supplemented with rifampicin (50 mg/l), kanamycin (50 mg/l), gentamycin (20 mg/l). A single colony was pre-cultured for 2 days in 3 ml YEB supplemented with antibiotics. A 20 µl aliquot was transferred into 10 ml YEB liquid medium containing antibiotics supplemented with 750 µl PCV (Packed Cell Volume) of 2-week old fresh EC of Thomson Seedless. The culture was grown overnight on an orbital shaker at 28°C and 200 rpm. When optical density (OD600) reached 0.8, the culture was centrifuged at 3500 g for 10 min and the pellet was re-suspended in MPM01 liquid medium, pH 5.2 to final OD600 0.3 and mixed with 10–15 calli (2–3 week-old) of 50 µl PCV in 2 ml-Eppendorf tubes. After 10 min on a gyratory shaker (at 20–22°C), EC were collected by blotting on filter paper, transferred (as 50 µl PCV aliquots) onto solid MPM01 supplemented with acetosyringone 100 µM and vacuum infiltrated (700 mbars, 5 min, twice). After 4 days culture at 20°C in the dark, EC were washed three times with MPM01 liquid medium supplemented with cefotaxim 600 mg/l, blotted dry and placed (as 50 µl PCV aliquots) onto MPM01 medium containing cefotaxim 600 mg/l. Plates were incubated in the dark, at 28°C, 80% RH.

### Selection and Regeneration of Transgenic Grape Plants

After 1–2 weeks culture, EC were transferred onto MPM1 medium supplemented with kanamycin 25 mg/l for GFP construction and BASTA® (50 mg/l) for GF-FG hairpin construction. EC treated the same way, without bacteria, were used as controls. EC were sub-cultured at three-week intervals onto fresh medium until GFP-fluorescent isolated sectors resistant to kanamycin or/and to BASTA® emerged. These sectors were picked and transferred onto fresh medium and plants were regenerated following the step-wise MPM series [17], [18], however with media supplemented with Cefotaxime 600 mg.l^−1^. Transgenic plants were maintained as cuttings on WPM supplemented with active charcoal (3 g/l) and Cefotaxime (300 mg.l^−1^).

### RNA Analysis

Total RNA was extracted from Grapevine and *Arabidopsis* tissues using the Tri-Reagent (Sigma, St Louis, MO) according to the manufacturer’s instructions. RNA gel blot analysis of low-molecular-weight RNA was performed using 15 µg of total RNA, as described previously [21]. Ethidium bromide staining of total RNA before transfer was used to confirm equal loading. Radio-labeled probes @GF and @P were made by random priming reactions in the presence of α-32P-dCTP (22). miR159, a stable, abundant and conserved miRNA in plants is used as loading control. Complementary DNA oligonucleotides were end-labelled with γ-32P-ATP using T4 PNK (New England Biolabs, Beverly, MA). After probing, the membranes were exposed to X-ray films.

The cDNA synthesis used the Invitrogen® kit, oligodT primers and 100–500 ng total RNAs out of which the equivalent of 20 ng cDNAs were used for qRT-PCR with 10 µl SsoFast™ EvaGreen® Supermix and 300 nM of each primer, V.v UBQ s GTGGTATTATTGAGCCATCCTT; V.v UBQ r AACCTCCAATCCAGTCATCTAC; for grapevine and; A.t ExP s GAGCTGAAGTGGCTTCCATGAC; A.t ExP r GGTCCGACATACCCATGATCC; A.t GAPDH 600b s AGGTGGAAGAGCTGCTTCCTTC; A.t GAPDH 600b r GCAACACTTTCCCAACAGCCT, for *Arabidopsis*. These reactions (95°C, 30 sec, 40 cycles 95°C, 5 sec and 60°C, 5 sec and finally 75°C, 10 sec for data acquisition with Arabidopsis and 80°C for grapevine) were performed in 96-well plates with CFX96TM Real-Time System C1000 Touch Thermal Cycler using SsoFastTM EvaGreen® Supermix. Data were analyzed using the BioRad Manager Software. For GFP transcript analysis, GFP s CAC-AAC-GTA-TAC-ATC-ATG-GCA-GAC, GFP r GAT-TGT-GTG-GAC-AGG-TAA-TGG-TTG-TC (95°C, 5 mn, 35 cycles 95°C, 30 sec and 60°C, 3 sec, 72°C 50 sec and finally 78°C, 10 sec for data acquisition) conditions were used. Relative transcript levels were calculated using the reference UBQ gene for grapevine and ExP (AT5G12240) or GAPDH600b (AT1G13440) for Arabidopsis according to Paffl [22]. Mean and deviations were calculated with data from three full experimental repetitions.

### Microscopy and Image Processing

Green-fluorescent-protein-derived expression in calli, embryos and developing plantlets were observed under a Nikon SMZ 1500 microscope (Nikon Corp., Tokyo, Japan) equipped with illumination from a Nikon UV lamp passed through a Nikon filter set with a 465–495 nm band pass excitation filter, a 505 nm dichroic mirror and a 510 or 535 nm band pass barrier filter. Photographs were taken with a Nikon 4.1 mega pixel digital camera attached to the microscope. To allow comparison, the magnification and acquisition characteristics were kept constant for the embryogenic calli, buds and the same stands true for the first leaf of all samples. Images were analyzed with ImageJ software version 1.46 (http://rsbweb.nih.gov/ij/). This consisted in measuring total intensities in green channel of acquired color images. Occasional pixels showing saturated values were excluded from the measurement area by thresholding. Intensities were normalized against measured area, yielding a mean grey value, and allowing comparison across different samples. Deviations were calculated from data obtained in 3 independent repetitions with 5–10 plants for each experiment. The data obtained are shown using value for the GFP line and the silenced line grown at 26°C as references.

## Results

### Transgenic Vines Carrying a GFFG Inverted-repeat Construct Produce GF siRNAs

**Figure 1:**
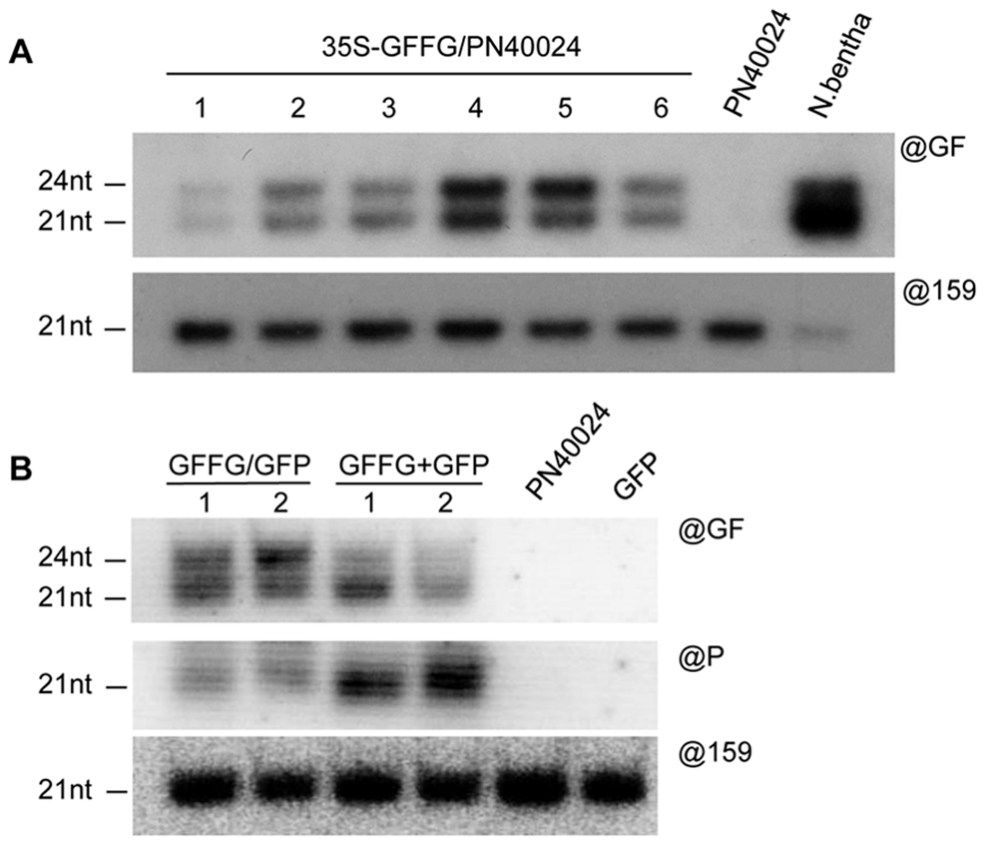
Efficient RNA silencing triggered by inverted-repeat constructs in grapevines. (**A**) Northern analysis of ‘GF’ siRNA (@GF) and miRNA (@159) accumulation in transgenic grapevines (PN40024 accession) expressing an inverted-repeat (IR) construct and in a GF-FG/N. Benthamiana line as control. (**B**) Northern analysis of ‘GF’ siRNA, ‘P’ secondary siRNA (@P) and miRNA (@159) accumulation in transgenic grapevines (PN40024 accession) expressing GFP either alone (GFP) or together with the GFFG IR construct.

As a first step towards implementing the best strategy to obtain virus-resistant transgenic grapevines, we first addressed whether transgenic vines undergo the first step of IR-PTGS, i.e. if they produce siRNAs from an inverted-repeat construct. To do so, we developed an efficient transformation procedure on the sequenced PN40024 accession, which derived from a near homozygous inbreeded Pinot Noir line obtained by successive selfings of the cultivated Pinot Noir (clone 162) [16]. The Embryogenic calli (EC) were initiated from somatic cells after anther filament cultures with an efficiency of 4.09±1.25% EC/anther plated (data not shown) and these EC lines could be maintained *in vitro* using established protocols [17], [18]. The friable EC were then transformed, following the improved procedure (see Material and Methods), to introduce an IR-construct expressing the 5′ part (‘GF’) of a GFP transgene, as an inverted-repeat, linked to a selectable marker conferring resistance to BASTA [23]. Six to eight months after the initial transformation, several BASTA-resistant transgenic true-to-type plants were regenerated, indicating that the PN40024 line is amenable to embryogenic callus induction and to stable transformation. Northern analysis of six independent GFFG transgenic grape lines showed that they all accumulated, although to various levels, both 21-nt and 24-nt siRNA corresponding to the ‘GF’ sequence (Figure 1A), indicating that dsRNA is similarly processed in *Vitis* than in the other plant species tested to date.

### GF siRNAs Induce IR-PTGS on a GFP Target Transgene and Transitive Production of P-derived Secondary siRNAs

The EC line GFFG#4, which accumulates the highest level of GF siRNAs (Figure 1A), was selected for a second round of transformation with a GFP transgene linked to a selectable marker conferring resistance to kanamycin. Double transgenic lines, hereafter referred to as GFFG/GFP, were selected based on their resistance to kanamycin. In parallel, wild-type EC were directly co-transformed with GFFG and GFP transgenes. Double transgenic plants, hereafter referred to as GFFG+GFP, were selected based on their resistance to both kanamycin and BASTA® Two independent GFFG/GFP lines and two independent GFFG+GFP lines were kept for further analysis.

Northern analysis revealed that all four selected lines accumulated both 21-nt and 24-nt siRNA corresponding to the ‘GF’ sequence (Figure 1B), as observed in the initial GFFG material (Figure 1A). Beside these so-called primary siRNAs, that are directly processed from the IR-construct, GFFG/GFP and GFFG+GFP lines also produced 21-nt, but not 24-nt, siRNAs from the GFP mRNA. This process coined ‘transitivity’, is involved in the amplification of the RNA silencing response through the activity of RDR6 on RNA fragments resulting from the cleavage of the GFP target mRNA by siRNAs derived from the GFFG construct [23]. The transitivity can be measured by the relative accumulation of secondary siRNAs with sequence of the non-overlapping 3′ part of the GFP transgene (‘P’) [19]. These secondary ‘P’ siRNAs were readily detectable in all GFFG/GFP and GFFG+GFP transgenic lines tested indicating the functionality of IR-PTGS in grapevine (Figure 1B). Of note, the levels of secondary siRNAs were lower in the GFFG/GFP plants compared with the GFFG+GFP transgenic lines. This most likely results from the very high amount of primary IR-derived siRNAs in the former that trigger an efficient clearance of the GFP mRNAs that are normally used as substrates by RDR6 to prompt production of secondary siRNAs.

### IR-PTGS is more Efficient in Somatic Tissues than in Actively Dividing Cells

**Figure 2:**
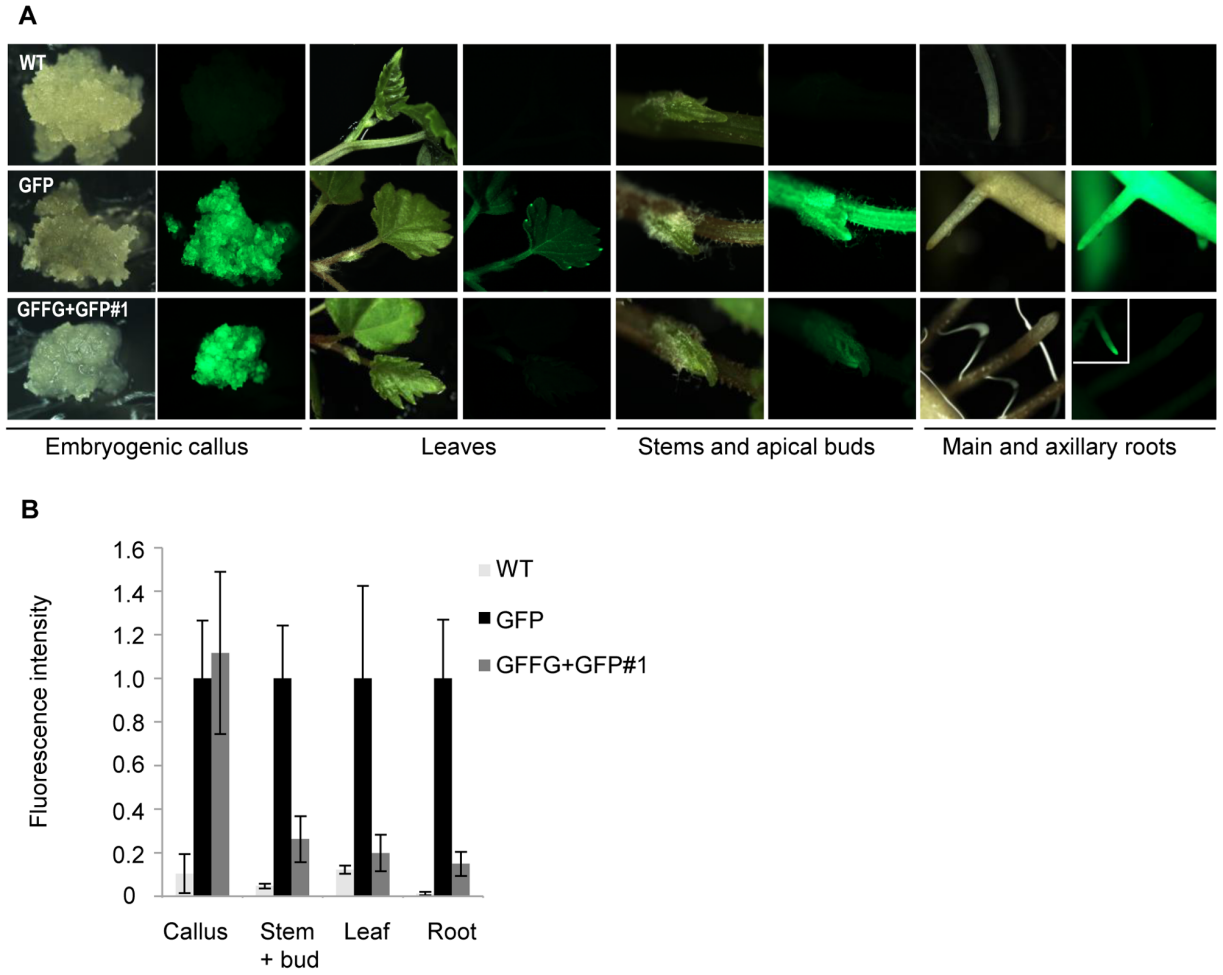
GFP silencing in grapevine tissues. (**A**) Silencing of the GFP is efficient in somatic tissues of GFFG+GFP transgenic grapevines but is impaired in meristematic tissues such as apical bud or occasional root apex and suppressed in calli. (**B**) Semi-quantitative analysis of the fluorescence in the tissues depicted in A. Fluorescence intensity is represented as grey level intensity/pixel. Means and standard deviations are calculated from at least 3 experimental repetitions of 5 different plants.

Observation under UV light revealed that EC from GFFG+GFP lines were as fluorescent as our control transgenic *Vitis* line expressing only the GFP transgene (Figure 2A). This observation is in agreement with previous results showing that cell dedifferentiation and proliferation induced by exogenous hormone treatments was shown to be sufficient to suppress GFP silencing in the growing calli [21]. However, upon regeneration of the GFFG+GFP grape lines, very low to undetectable levels of GFP were observed in all somatic tissues examined such as leaves, stems and main as well as axillary roots. In contrast, all tissues of transgenic GFP grape lines remained bright green fluorescent (Figure 2A). This was confirmed by quantification of the fluorescence signal (Figure 2B) which showed a 5-fold decrease of flurorescence in stem, leaf and root tissues of the silenced lines, when compared to GFP line. This plant material, either maintained by in vitro cuttings or transferred to soil kept all its characteristics. Similar observations were obtained when analyzing 8 independent GFFG+GFP or GFFG/GFP transgenic grape lines over a 1.5 year period (data not shown). Of note, a faint, but visible, GFP expression level was observed in the apical meristems, in axillary buds and, on occasions, in the tip of some rootlets of the GFFG/GFP or GFFG+GFP lines (Figure 2A and data not shown). Thus, as previously observed in other plant species, IR-constructs trigger in *Vitis* a potent RNA silencing reaction in somatic tissues resulting from dsRNA processing and transitivity but is, somehow, less efficient in actively dividing cells [21], [24].

### IR-PTGS is Functional in Grape, but not Arabidopsis, Plants Grown at Low Temperatures

**Figure 3:**
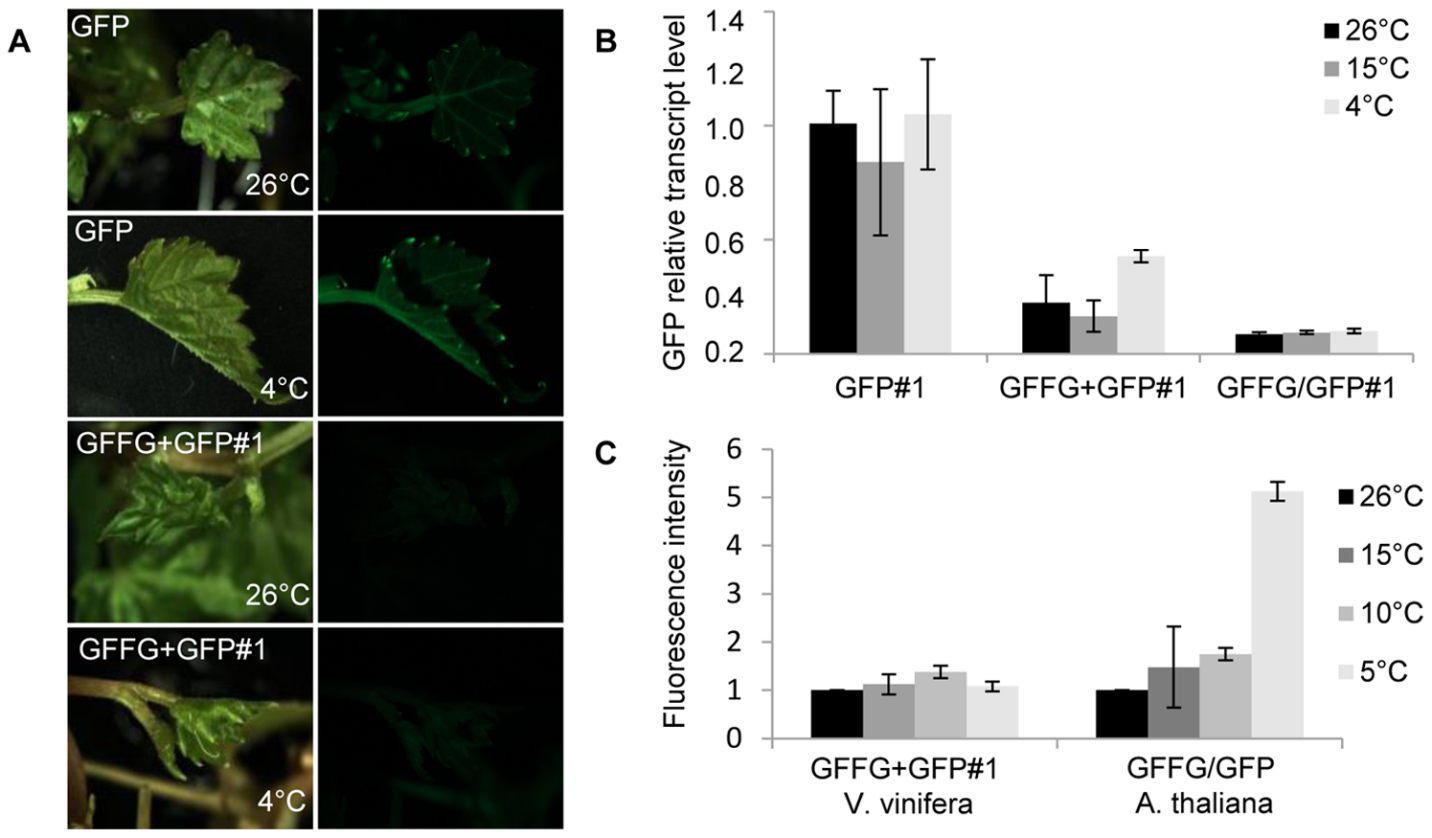
Silencing of the GFP is maintained in grapevines grown at low temperatures. (**A**) Silencing of the GFP is maintained in somatic tissues of GFFG+GFP transgenic grapevines grown at 4°C. (**B**) Quantitative real-time PCR of the GFP mRNA in GFP expressing or GFP-silenced grapevines grown at 26°C, 15°C or 4°C. mRNA levels were normalized to that of the reference ubiquitin gene for grapevine. (**C**) Semi-quantitative analysis of the fluorescence in GFFG+GFP transgenic grapevines or GFFG/GFP *Arabidopsis* grown at 26°C, 15°C, 10°C or 4°C. Fluorescence intensity is represented as grey level intensity/pixel. Means and standard deviations are calculated from at least 3 experimental repetitions of 5 different plants.

RNA silencing in *Arabidopsis thaliana*, *Nicotiana benthamiana* and *Solanum tuberosum* was previously shown to function at 20–26°C but not at 15°C [12], [13]. In contrast to these herbaceous species, perennial cultivated grapevines and wild *Vitis* species are often exposed in vineyards, or in their natural habitat, to far lower temperatures than this 15°C breaking-point. Therefore, to assess if the IR-PTGS approach can be implemented in the field to trigger efficient virus resistance in *Vitis*, we next assessed the temperature-sensitivity of RNA silencing in this species. To do so, after a 3–4 weeks growth period at 26°C, untransformed (WT) and transgenic GFFG+GFP, GFFG/GFP and GFP grape plantlets were transferred for another 4–8 weeks cultivation period at 26°C, 15°C, 10°C or 4°C. Similar experiments were also conducted with a transgenic *Arabidopsis* expressing the same GFP and GF-IR transgenes [21]. Although these low temperatures slowed down the growth of both *Arabidopsis* and *Vitis*, plants continued to develop and initiate new leaves. In *Arabidopsis*, we observed a gradual increase of the fluorescence, notably in roots, and finally in newly formed leaves, reaching a 5-fold increase of fluorescence as shown by quantification of the fluorescence intensities in plant grown at 4°C (Figure 3C).

**Figure 4:**
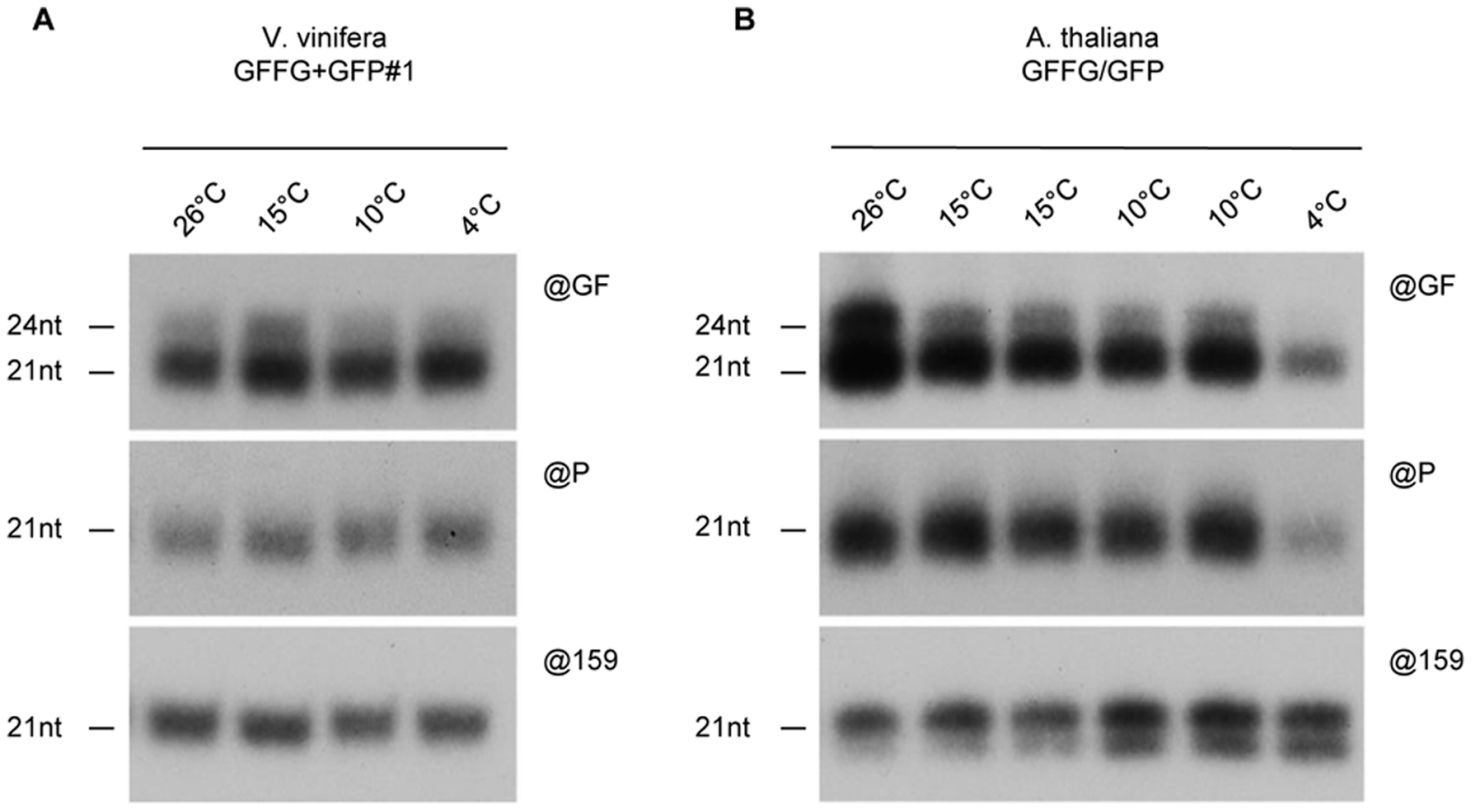
siRNA levels are not altered in low-temperature grown grapevines. Northern analysis of ‘GF’ siRNA (@GF), ‘P’ secondary siRNA (@P) and miRNA (@159) accumulation in GFFG+GFP transgenic grapevines (**A**) or GFFG/GFP Arabidopsis (**B**) grown at various temperatures.

Interestingly, in the newly formed leaves of GFFG/GFP and GFFG+GFP *Vitis* transgenic lines, GFP silencing was maintained, even at temperatures as low as 4°C (Figure 3A). These observations were confirmed by quantitative RT-PCR of the GFP mRNA (Figure 3B). We ruled out that the low GFP level was due to an inhibition of the 35S-driven GFP transgene transcription rate, in response to cold treatment, as both GFP fluorescence and mRNA steady-state levels were high in the GFP-expressing transgenic grape lines and remained steady from 26°C to 4°C (Figure 3A–C). Northern analysis revealed that neither the ‘GF’ nor the ‘P’ siRNA accumulation were altered by low temperatures in the silenced GFFG/GFP and GFFG+GFP *Vitis* transgenic lines, further supporting that RNA silencing, at least when triggered by IR constructs, operates efficiently in *Vitis* at temperatures as low as 4°C (Figure 4A). By contrast, GFP silencing in *Arabidopsis*, triggered by the same IR-construct, was released at 4°C(Figure 3C) and this inhibition was correlated with a strong decrease in both ‘GF’ and ‘P’ siRNA accumulation at this temperature (Figure 4B). Remarkably, IR-PTGS and siRNA production do not seem to be affected in *Arabidopsis* grown at 15°C or even at 10°C (Figure 3C and 4B), suggesting that, as opposed to S-PTGS which was already hindered at these temperatures [12], [13], IR-PTGS is more resistant to low temperature, in agreement with previous observations [29].

## Discussion

IR-PTGS, which is triggered by constructs directly expressing dsRNA, is more efficient than S-PTGS, which is triggered by constructs expressing aberrant single-stranded RNA that first need to be converted to dsRNA [25]. As a result, IR-PTGS has already been successfully used to elicit strong antiviral defence in many plant species [23], [26]. To determine if IR-PTGS functions in a conserved manner in grapevines, we transformed the sole inbred grapevine whose genomic sequence is accessible. Despite considerable and long-lasting efforts, it is well-known that the grape genus remains rather recalcitrant to stable transformation, with the exception of few accessions for which the best transformation efficiencies to be expected range from 1–33 transgenic plant/g of embryogenic callus (EC) treated [27]. Even though Pinot Noir is reputed for being refractory to *in vitro* culture [17], [18], [27], the transformation procedure developed here on the PN40024 line allowed us to routinely produce between 10–100 independent transgenic plant/g of EC (Table 1 and data collected by our open-to-all transformation platform over a three-year period; plateforme-transfovigne@colmar.inra.fr).

**Table 1:** 

Genotype	Constructs	Number ofexperiments/ECinoculated	Mass of ECinoculated in g	Number of transgenicEC obtained	Transformationefficiency
PN40024	GFP	3/30	3	>300	100
PN40024	GF-FG	2/15	1.5	9	6
PN40024	GF-FG+GFP	2/13	1.3	13	10
GF-FG lines	GFP	5/47	4.7	142	30.21

Transformation efficiency = number of transgenic EC/gram of Embryogenic Callus according to Bouquet et al. [27]. 1 embryogenic callus of 100 µl PCV.

The efficient transformation procedure developed here (Table 1) and the efficiency of IR-PTGS observed in the transformants, not only open this species to “fast” reverse genetic studies for basic or applied research but also pave the way towards implementing the best strategy to generate silencing-based virus-resistant *Vitis*. Indeed, our results collectively indicate that IR-constructs trigger a potent and low temperature-resistant RNA silencing reaction in this species. This unaltered RNA silencing activity at low temperatures in grapevine probably helps this perennial plant, which undergo cold stress every winter, but also during spring time, when green arms develop from the latent buds, to cope with pathogens all along the year. Efficient IR-PTGS relies on the activity of several different RNA silencing factors. These include DCL4 for production of both primary and secondary siRNAs, AGO1 for their action and RDR6 for their amplification. Therefore, the observed sensitivity of IR-PTGS to low temperature in *Arabidopsis* or tobacco suggests that, in these later, as opposed to *Vitis*, one or several of these factors is/are somehow impaired [12]. This will certainly deserves careful examination in the future.

Altogether, our data suggest that the breaking point of silencing is below 4°C for grapevine, in contrast to 15°C for *Arabidopsis*, *N. benthamiana*, potato, *N. tabacum*, soybean and petunia [12], [13], [14], [15]. Remarkably, other kingdoms also exhibit a temperature-dependent breaking point of silencing: 22°C in drosophila [28] and 27°C in mammalian cells [29], suggesting that RNA silencing has adapted to the environment of each species. Whether other perennial plants, such as poplar or fruit trees, exhibit similar unaltered RNA silencing activity at low temperature remains to be addressed.

Despite the efficiency of IR-PTGS in most grapevine tissues, it should be noted that occasional GFP fluorescence was observed in the apical region of some rootlets (Figure 2A). Interestingly, the *Xiphinema index* nematodes, which are specific vectors of GFLV, perforate the grape roots at this particular 1–2 mm rootlet region. After a first attack, such feeding zones become then attractive to other nematodes where they feed and reproduce [30]. Therefore, we cannot yet fully exclude that, upon feeding, *X. index* can inject GFLV particles into those scarce and silencing-defective rootlet tissues where it could replicate and thus build up a reservoir for possible disease spread in the entire plant, being in a way Achilles’ heel of vine.
